# Phylogeny, Resistome, and Virulome of *Escherichia coli* Causing Biliary Tract Infections

**DOI:** 10.3390/jcm8122118

**Published:** 2019-12-02

**Authors:** Ángel Rodríguez-Villodres, Rémy A. Bonnin, José Manuel Ortiz de la Rosa, Rocío Álvarez-Marín, Thierry Naas, Javier Aznar, Jerónimo Pachón, José Antonio Lepe, Younes Smani

**Affiliations:** 1 Clinical Unit of Infectious Diseases, Microbiology and Preventive Medicine, University Hospital Virgen del Rocío, 41013 Seville, Spain; anrovi1797@gmail.com (A.R.-V.); ortiz_dela_8@hotmail.com (J.M.O.d.l.R.); jaznar@us.es (J.A.); jalepe@cica.es (J.A.L.); 2 Institute of Biomedicine of Seville (IBiS), University Hospital Virgen del Rocío, CSIC, University of Seville, 41013 Seville, Spain; 3 LabEx Lermit, EA7361, Université Paris-Sud, Université Paris-Saclay, 91190 Saint-Aubin, France; remy.bonnin@u-psud.fr (R.A.B.); thierry.naas@aphp.fr (T.N.); 4 The Evolution and Ecology of Resistance to Antibiotics” Unit, Institut Pasteur-APHP-Université Paris Sud, 91400 Orsay, France; 5 Bacteriology Hygiene Unit, APHP, Hôpital Bicêtre, Le Kremlin-Bicêtre, 94270 Paris, France; 6 Department of Microbiology, University of Seville, 41009 Seville, Spain; 7 Department of Medicine, University of Seville, 41009 Seville, Spain

**Keywords:** *Escherichia coli*, biliary tract infection, virulome, resistome, biofilm

## Abstract

*Escherichia coli* is the most frequent Gram-negative bacilli involved in intra-abdominal infections. However, despite high mortality rates associated with biliary tract infections due to *E. coli*, there is no study focusing on this pathogen. In this study, we have characterized a group of 15 *E. coli* isolates obtained from 12 patients with biliary tract infections. Demographic and clinical data of the patients were recovered. Phylogeny, resistome, and virulome analysis through whole genome sequencing and biofilm formation were investigated. Among the 15 *E. coli* isolates, no predominant sequence type (ST) was identified, although 3 of them belonged to unknown STs (20%). Resistance to ampicillin, amoxicillin/clavulanic acid, cotrimoxazole, and quinolones was more present in these isolates; whereas, third and fourth generation cephalosporins, carbapenems, amikacin, tigecycline, and colistin were highly active. Moreover, high diversity of virulence factors has been found, with *sfa*, *fimH*, and *gad* the most frequently detected genes. Interestingly, 26.6% of the *E. coli* isolates were high biofilm-producers. Altogether, our data characterized for the first time *E. coli* isolates associated with biliary tract infections in terms of genomic relationship, resistome, and virulome.

## 1. Introduction

*Escherichia coli* is a Gram-negative bacterium with high clinical relevance. This microorganism may cause severe community- and hospital-acquired infections including bacteraemia, urinary tract, respiratory, and intra-abdominal infections [1]. Among intra-abdominal infections, acute cholangitis, an infection of the biliary system, is usually associated with high morbidity and mortality (5–13%) [2,3], reaching up to 29% in cases of malignant obstruction [3]. In case of biliary tract infection, *E. coli* was reported to be the most frequent pathogen isolated from bile samples (23%) [4].

*E. coli* sequence type 131 (ST131) identified by multilocus sequence typing (MLST) has been reported as the most prevalent clonal group worldwide, frequently associated with multidrug-resistance (MDR) and infections [5]. Other pandemic lineages such as ST69, ST95, and ST73 have been also associated with more virulent *E. coli* isolates [6,7]. Focusing at the abdominal level, the phylogenetic group B2 of *E. coli* non ST131 has been described in inflammatory bowel disease [8]. However, the epidemiology of *E. coli* causing biliary tract infection is poorly documented. 

*E. coli* needs to move from the large intestine to the biliary tract to produce a biliary tract infection. This process depends on virulence factors that are usually located in pathogenicity islands and can be divided into five main groups: 1) adhesins, 2) toxins, 3) siderophores, 4) capsular, and 5) protectins and invasins [9]. Thus, some genes coding for virulence factors have been associated with urinary tract infections and bacteraemia such as *pap, fimH*, *sfa*, *iha*, *bfp* (adhesins), *hlyA*, *cnf1*, *sat* (toxins), *fyuA*, *iutA* (siderophores), *kpsMTII* (capsule) and *aer*, *traT*, *ompT*, *usp*, and *malX* (miscellaneous proteins) [10,11,12,13,14]. However, to our knowledge, only limited studies investigated the microbiological features of *E. coli* causing biliary tract infections [15,16], despite the fact that *E. coli* is the most frequent etiological agent [17]. Characterizing the virulence factors that facilitate the establishment of biliary tract infections in *E. coli* may be helpful in order to identify potential targets that could be locked as a therapeutic strategy. To this end, we examined the clonal relationship, the resistome, and virulome in 15 *E. coli* isolates responsible for biliary tract infections in Spain.

## 2. Materials and Methods

### 2.1. Bacterial Strains

Fifteen *E. coli* isolates obtained from bile and blood of 12 patients with biliary tract infection (BTI), and 21 isolates of 20 patients with non-BTI (bacteremia and non-biliary intraabdominal infections) hospitalized at the University Hospital Virgen del Rocío (Seville, Spain) were included in this study. The isolates were collected at the Clinical Microbiology Service from blood and/or bile samples of these patients and then, stored at −80 °C in Luria Bertani (LB) broth supplemented with 30% glycerol. Identification of the isolates was performed using MALDI-TOF (Bruker Daltonik GmbH, Leipzig, Germany), as described previously [18] and whole genome sequencing (WGS) (HiSeq systems, Illumina, USA). *E. coli* ATCC 10536 strain was used as the positive control for biofilm formation. *E. coli* ATCC 25922 was used as the control strain for antibiotic susceptibility testing.

### 2.2. Demographics, Clinical Data, and Follow Up

The diagnosis of the biliary tract infection was made by members of the Infectious Disease Service and/or the Intensive Care Unit (ICU), according to the defined criteria [19]. The following variables were collected from the 12 BTI and 20 non-BTI patients: age and gender, Charlson score, an index that categorize comorbidity of patients based on the International Classification of Diseases diagnosis code [20] and McCabe score, a score that can obtain comparisons regarding the importance of host factors based on the severity of the underlying disease [21], the acquisition type of the BTI and non-BTI (community, healthcare-associated, or hospital), severity (sepsis or septic shock) [22], antibiotic exposure in the previous 2 months of hospitalization and duration of the antimicrobial treatment. The patients were followed until hospital discharge, death, or 30 days, which ever occurred first. The study was approved by the Ethics Committee of the University Hospital Virgen del Rocío and University Hospital of Virgen Macarena of Seville (approval no. 0023-N-16, 01/10/2016). Written informed consent was signed by all patients before inclusion in the study.

### 2.3. Antimicrobial Resistant Profile

The minimum inhibitory concentration (MIC) was determined by broth microdilution method using MicroScan Walk Away NM44 panels (Beckman Coulter, Inc., USA) for the following antibiotics: ampicillin, amoxicillin-clavulanic acid, piperacillin-tazobactam, cefuroxime, cefotaxime, ceftazidime, cefepime, imipenem, meropenem, ciprofloxacin, levofloxacin, gentamicin, tobramycin, amikacin, cotrimoxazole, and tigecycline. Colistin was tested by standard broth microdilution as recommended by the European Committee of Antimicrobial Susceptibility Testing (EUCAST) [23] and the interpretation criteria was performed following the EUCAST breakpoints [24]. MDR criteria were established as non-susceptible to ≥1 agent in ≥3 antimicrobial categories [25]. *E. coli* ATCC 25922 was used as the control strain.

### 2.4. Whole Genome Sequencing

Genomic DNA was extracted using an UltraClean microbial DNA isolation kit (MO BIO Laboratories, Mo-Bio, Sait-Quentin en Yvelines, France) from overnight cultures in LB agar (Bio-Rad, Marnes-la-Coquette, France). Genomic DNA quantification was performed using a Qubit fluorometer (Life Technologies, Carlsbad, CA, USA) and adjusted to 0.2 ng/μL. The DNA libraries were prepared using the NexteraXT v3 kit (Illumina, San Diego, CA, USA), according to the manufacturer’s instructions, and then run on the HiSeq systems (Illumina, USA) to generate paired-end 150-bp reads. De novo assembly of Illumina reads was performed using CLC genomic workbench 10.1 according the manufacturer’s recommendations (Qiagen, Courtaboeuf, France). The genome was annotated using the Rapid Annotations using Subsystems Technology (RAST) tool. The acquired antimicrobial resistance genes were identified by uploading assembled genomes to the Resfinder server v2.1 (http://cge.cbs.dtu.dk/services/ResFinder-2.1) [26]. Virulence genes were searched using https://cge.cbs.dtu.dk/services/VirulenceFinder/.

### 2.5. Phylogenetic Analysis

Sequence alignment and phylogenetic trees were performed using the software MEGA7 and Evolview online tool [27,28]. Neighbor-joining trees were built from concatenated sequences of the 7 housekeeping genes (*adk*, *fumC*, *gyrB*, *icd*, *mdh*, *purA*, and *recA)* used previously in MLST assay [29], and obtained for whole genome sequencing analysis. The evolutionary distances were computed using Kimura’s two-parameter model with gamma-distributed rate variation of 0.8 [30]. A bootstrap consensus tree inferred from 1000 replicates was depicted to represent the evolutionary history of the taxa analyzed [31].

### 2.6. Biofilm Formation Assay

An abiotic solid surface biofilm formation assay was performed as described previously [32]. In brief, overnight cultures of the clinical *E. coli* isolates were diluted 1:100 in fresh LB broth in 96 well plates without shaking and incubated at 37 °C for 48 h. Biofilm was stained with crystal violet 0.4% (v/v) and quantified at 580 nm after solubilization with ethanol 95%. *E. coli* isolates were classified as biofilm-formers if they yielded optical density at 580 nm (OD_580nm_) values that were at least twice those of the negative control. *E. coli* ATCC 10536 strain was used as positive control.

### 2.7. Statistical Analysis

A descriptive analysis was performed for demographics and clinical variables of the patients included in the study, with median and interquartile range for the quantitative variables and frequency distribution (%) for the qualitative variables. Fisher and χ^2^ tests were used for categorical variables and continuous variables were analyzed using 2-sample t test or Mann Whitney U test. For biofilm formation assay, the group data are presented as mean ± SEM. Differences were considered significant at *p* < 0.05. All statistical analyses were performed using SPSS software, version 23.0 (IBM Corporation, Somers, New York, USA).

### 2.8. Nucleotide Sequence Accession Number

The WGS of the *E. coli* isolates generated in the study were deposited in GenBank under the BioProject accession number PRJNA557044.

## 3. Results

### 3.1. Bacterial Isolates 

MALDI-TOF identified the 15 bacterial isolates from the 12 patients with bile infections, following the clinical criteria established by Solomkin et al. [19], as *E. coli.* Among them, 11 (73.3%) were isolated from bile samples and 4 (26.6%) from blood cultures. Three patients had two isolates each: 140-HE and 23-AE from blood and bile cultures, 43-HE and 4-AE from blood and bile cultures, and 60-AE and 61-AE from the bile culture, respectively.

### 3.2. Demographics and Clinical Data Analysis

Demographic and clinical features of the 12 and 20 patients with biliary tract infections (BTI) and non-BTI, respectively, included in this study are detailed in Table 1. To summarize, for both group of patients, the demographic (age and gender), comorbidities (Charlson and McCabe scores), sepsis or septic shock, and previous treatment and days of treatment were not significantly different. Regarding to the acquisition of the infection, significant difference was observed between both groups (*p* = 0.035). Importantly, there was no difference in mortality between both groups. 

### 3.3. Antimicrobial Resistance Profile

Antimicrobial susceptibility testing results are shown in Table 2. Non-significant difference has been observed between BTI and non-BTI isolates. The most frequent resistance found in BTI isolates was by far Ampicillin (73.3%), followed by amoxicillin-clavulanic acid (26.6%), cotrimoxazole (26.6%), and fluoroquinolones (13.3%). The rest of the antibiotics (third and fourth generation cephalosporins, carbapenems, amikacin, tigecycline, and colistin) were highly active against these *E. coli* isolates. For the non-BTI isolates, the most frequent resistance was by far Ampicillin (90.4%), followed by fluorquinolones (33.33%), amoxicillin-clavulanic acid (28.6%), cotrimoxazole (28.5%), and cefuroxime (14.3%). The rest of the antibiotics were highly active. 

A MDR (resistance to beta-lactams including or not cephalosporins, beta-lactams/beta-lactamases inhibitors, fluoroquinolones, cotrimoxazole, and/or aminoglycoside) pattern was found in 2 and 5 BTI and non-BTI isolates (13.3% and 23.8%), respectively. Extended spectrum beta-lactamase (ESBL) was only detected in the 2 non-BTI isolates; whereas, carbapenemase production were not detected in any of the BTI and non-BTI isolates.

### 3.4. Genetic Characterization

#### 3.4.1. Epidemiology

The WGS analysis of the *E. coli* isolates showed that 12 isolates belonged to 10 different STs and 3 isolates (3-AE, 47-HE, and 61-AE) belonged to novel STs (Table 3). Moreover, the isolate pairs recovered from the same patient 140-HE and 23-AE, and 43-HE and 4-AE belonged to ST131 and ST58, respectively. In contrast, the isolate pair 60-AE and 61-AE corresponded to two different STs, namely ST542 and an unknown ST, respectively. 

**Figure 1:**
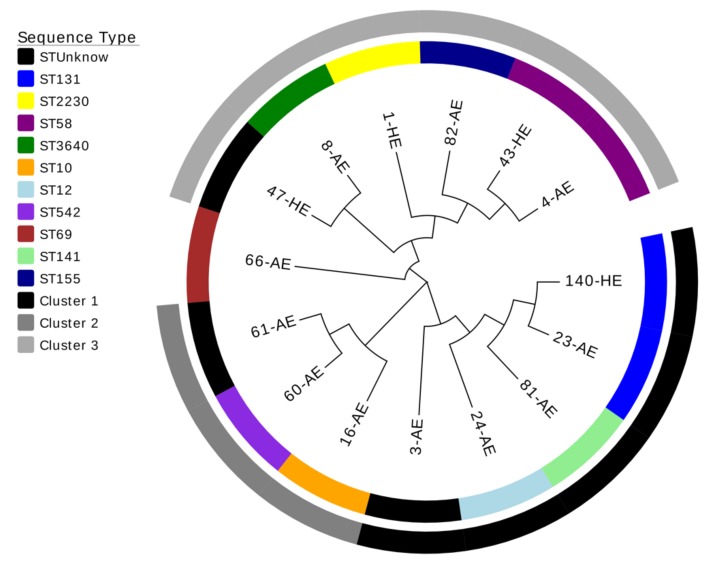
Phylogenetic tree using neighbor-joining method with 1000 bootstrap replicas of the 15 *E. coli* isolates from the patients with biliary tract infections.

The phylogenetic analysis of the concatenated sequences of the seven MLST housekeeping genes of each isolate showed a tree consisting of three clusters and one branch formed by only one isolate that belonged to ST69 (66-AE) and unrelated phylogenetically to the other isolates. The cluster 1 contained 5 isolates; two of them (140-HE and 23-AE) belonged to ST131 and 1 (3-AE) to an unknown ST related to ST12 complex. The cluster 2 contained 3 isolates; one of them (61-AE) belonged to an unknown ST related to ST542. Finally, the cluster 3 had 6 isolates; one of them (47-HE) belonged to an unknown ST related to ST3640 (Figure 1).

#### 3.4.2. Virulome

The WGS analysis has identified several genes coding for virulence factors in the *E. coli* isolates (Table 4). The most frequently detected genes were *sfa* (S fimbrial adhesin, 93.3%), *fimH* (type 1 Fimbriae, 93.3%), and *gad* (glutamate decarboxylase, 86.6%), followed by *lpfA* (long polar fimbria, 60.0%), *iss* (increased serum survival, 53.3%), *iroN* (enterobactin siderophore receptor protein, 53.3%), *iutA* (ferric aerobactin receptor, 53.3%), *mchF* (ABC transporter protein, 46.6%), *mchC* (microcin H47 maduration system, 20.0%), *senB* (enterotoxin, 20.0%), *mchB* (microcin H47, 13.3%), *astA* (heat-stable enterotoxin-1, 13.3%), *iha* (irgA homologue adhesion, 13.3%), *mcmA* (microcin M, 13.3%), *vat* (vacuolating autotransporter toxin, 13.3%), *papG* (P fimbrial adhesin, 13,3%), *tsh* (temperature-sensitive hemagglutinin, 6.6%), *air* (enteroaggregative immunoglobulin repeat protein, 6.6%), *eilA* (Salmonella *hilA* homologue, 6.6%), *ireA* (siderophore receptor, 6.6%), *cnf1* (cytotoxic necrotizing factor-1, 6.6%), and *ipaH* (invasion plasmid antigen, 6.6%). Other relevant virulence factors in *E. coli* such as *hlyA* (hemolysin A), *stx-1* and *stx-2* (Shiga toxins-1 and -2), *cdtB* (cytolethal distending toxin), *eaeA* (intimin), and *bfp* (bundle-forming pilus) were not found in these isolates.

#### 3.4.3. Resistome

The WGS revealed that the *bla*_TEM-1a_ and *bla*_TEM-1b_ genes, coding for the broad spectrum beta-lactamase TEM-1, were the most frequently detected enzyme (66.6%). Mutations in *gyrA*, *parC*, and *parE* genes, associated with fluoroquinolone resistance, were detected in 33%, 6.6%, and 20% of the isolates, respectively. Resistance to aminoglycosides (*aph*, 40%; *aadA*, 33.3%; and *strA*, 13.3%), sulphonamides (*sul*, 46.6%), trimethoprim (*dfrA,* 33.3%), tetracycline (*tet*, 20.0%), azithromycin (*mph*, 13.3%), and chloramphenicol (*catA*, 13.3%) were also detected in these isolates (Table 5).

#### 3.4.4. Biofilm Formation

**Figure 2:**
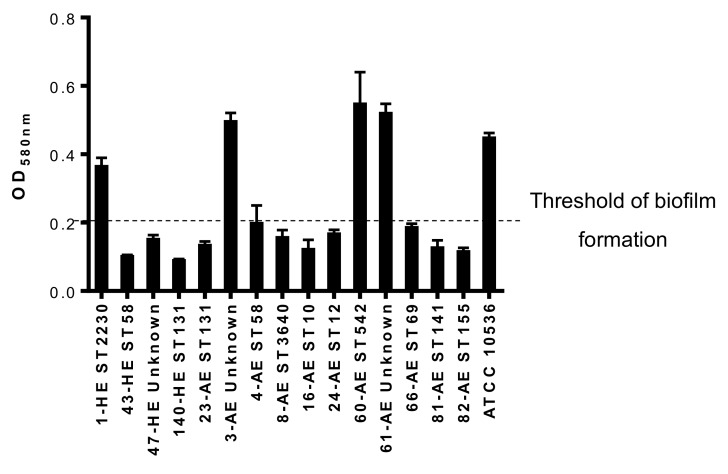
Biofilm formation by the 15 *E. coli* isolates isolated from patients with biliary tract infection. Unknown: unknown ST.

The analysis of the biofilm formation in abiotic surface showed that 4 isolates (26.6%) were biofilm-producers with OD_580nm_ higher than 0.2. Two of them belonged to unknown ST (3-AE and 61-AE), one to ST2230 (1-HE), and one to ST542 (60-AE) (Figure 2).

## 4. Discussion

This study shows, for the first time, the genomic relationship, resistome, and virulome of 15 *E. coli* isolates obtained from bile and blood cultures of 12 patients with biliary tract infection. Twenty percent of *E. coli* isolates belonged to unknown ST but with different allele combinations. This percentage is higher than those found in other areas of infections such as urinary tract infection (3.5%), bloodstream infection (10.5%), and left-sided inflammatory bowel disease (0%) [6,8,33]. Of note, twenty-one *E. coli* isolates recovered from different non-biliary sources in our hospital and over the same period of time were also characterized phylogenetically by MLST and none of them belonged to unknown STs (Table S1).

Although, ST131, ST95, ST73, and ST69 are clearly predominant STs in human *E. coli* infections such as urinary tract or bloodstream infections [7,34], in our study only 2 *E. coli* isolates (140-HE and 23-AE, from the same patient) belonged to ST131 and another one (66-AE), to ST-69. The rest of the isolates belonged to other STs, which suggests, even though the sample size of this study is small, that in *E. coli* biliary tract infections, there is not a predominant ST. It is noteworthy to mention that 140-HE and 23-AE isolates that belonged to ST131 had similar resistance profiles (resistance to ampicillin, amoxicillin-clavulanic acid, and cotrimoxazole) with exception for piperacillin-tazobactam. Indeed, the isolates 140-HE and 23-AE were categorized susceptible to piperacillin-tazobactam by MicroScan and resistant to piperacillin-tazobactam by broth microdilution method. This could be due to the presence of different heteroresistant populations that cannot be detected by the automated Microscan system [35]. 

Regarding the antimicrobial resistance profiles of the 15 *E. coli* isolates of this study, ampicillin showed the highest percentage of resistance (73.3%), followed by amoxicillin-clavulanic acid (26.6%), cotrimoxazole (26.6%), and fluoroquinolones (13.3%). These data are in agreement with those of WGS analysis (Table 5). Specifically, *bla*_TEM_ genes involved in ampicillin resistance have been found in 66.6% of the isolates, *dfrA* gene involved in trimethoprim resistance has been found in 33.3% of the isolates, whereas *gyrA* and *parE* were more prevalent (33% and 20%) than the observed fluoroquinolones resistance rate (13.3%). However, Razaghi et al. reported that 54%, 31.8%, 22.7%, and 0% of *E. coli* isolated from bile presented resistance to ciprofloxacin, meropenem, ceftazidime, and amoxicillin-clavulanic acid, respectively [15]. These differences are likely the result of differences in local epidemiology. In Spain, the analysis of a total of 1429 *E. coli* isolates causing intra-abdominal infections in a surveillance study called SMART (Study for Monitoring Antimicrobial Resistance Trends) between 2016 and 2017 revealed that ciprofloxacin and amoxicillin-clavulanic acid presented resistance rates of 27.6% and 18.5%, respectively [36]. However, in Iran, the MDR pattern was detected in 95.5% of the *E. coli* recovered from bile, reporting different antimicrobial resistance rates in comparison with our results, mainly in the case of third generation cephalosporins (82% vs. 0%), carbapenems (≈70% vs. 0%), gentamicin (36.4% vs. 6.6%), and ciprofloxacin (45.5% vs. 13.3%), respectively [37]. 

The WGS showed a high diversity in the virulome among the *E. coli* isolates analyzed in this study, similarly to Fernández-Romero et al. observations [38]. None of the virulence factors genes detected were present in all of the isolates, indicating the absence of a unique gene essential for the development of *E. coli-*borne biliary tract infections. However, three of the virulence factors were detected in around 90% of the isolates: *sfa* and *fimH* that are involved in the adhesion to the cells and facilitate the penetration of bacteria into the tissues [39], and *gad,* which expression is relevant for the maintenance of *E. coli* in acid environments such as the biliary tract [40]. These data suggest that adhesion ability and resistance to bile acids seem to be important to produce a biliary tract infection. Transcriptomic confirmatory analyses will help to determine the involvement of these genes in the infections development, especially in the patients with two isolates recovered from bile and blood, respectively, in which the expression of these genes may change during the course of the infection. Similar data have been reported by Wang et al. [16] who studied the role of virulence factors in the development of *E. coli* bacteraemia in patients with acute cholangitis. They detected the presence of *fimH* and *iutA* in 88% and 50% of the isolates, respectively; whereas, *sfa* was detected only in 8% of the isolates [16]. Another study reported that *bfp* and *astA* were the most frequent virulence factors in *E. coli* isolated from bile [15]; although they did not analyze the presence of *fimH, sfa*, or *gad* in their isolates. It is important to mention, that no transcriptomic analyses were performed in this study to confirm the degree of expression of these virulence genes, even though they might be or not be expressed. 

Some genetic interconnections have been found between different isolates. It appears that the isolate 3-AE could be the ancestor of the cluster 1. Some virulence genes (*iss*, *ipfA*, *mchC*, and *mchB*) present in this isolate were lost when compared with the rest of the isolates of the cluster 1. In contrast, other virulence genes (*gad*, *mcmA*, *vat*, and *iutA*) were acquired in the rest of the isolates of the cluster 1. Similarly, we found that the isolate 60-AE acquired the genes *iss*, *ipfA*, *iroN*, *astA*, *senB*, *iha*, and *iutA*, when compared with the isolate 61-AE recovered from the same patient. In the same line, the isolate 47-HE, closely related to the isolate 8-AE, acquired the genes *astA* and *fimH*, when compared with the isolate 8-AE. 

Notably, we report, here, that the *E. coli* isolates do not only harbor virulence and resistance genes, but also 26.6% of them were high biofilm-formers. However, the relationship between biofilm formation and virulence or resistance pattern was not observed, and needs inclusion of more isolates in the future.

## 5. Conclusions

This study characterized, for the first time by WGS, the molecular epidemiological traits, virulome, and resistome of a collection of *E. coli* isolates from patients with biliary tract infections. Low proportion of *E. coli* ST131 was found and most *E. coli* isolates belonged to different STs. Antimicrobial susceptibility testing and WGS matched perfectly. Resistance to penicillins, cotrimoxazole, and fluoroquinolones were the most frequently encountered resistances. Furthermore, a high diversity of virulence factors was evidenced, with however, a predominance of genes involved in adhesion and resistance to biliary acids. A better knowledge of the microbiological features of *E. coli* causing biliary tract infection is important to improve the diagnosis and treatment of these patients.

## Figures and Tables

**Table 1:** Demographics and clinical data from patients with biliary tract infections (BTI) and non-BTI by *E. coli.*

Variable	BTI Patients (n = 12)	Non-BTI Patients (n = 20)	*P-*Value
Age, median (range)	64 (58–72.5)	64.5 (55.75–77.75)	0.893
Gender (female), n (%)	7 (58.3)	10 (50.0)	0.647
Charlson Score, median (range)	1.5 (0–3.75)	3 (0.25–6)	0.146
McCabe Score ultimately or rapidly fatal, n (%)	2 (16.6)	11 (55.0)	0.062
Acquisition, n (%)			
Community	9 (75.0)	6 (30.0)	**0.035**
Healthcare	2 (16.6)	5 (25.0)	
Nosocomial	1 (8.3)	9 (45.0)	
Sepsis or septic shock, n (%)	3 (25.0)	10 (50.0)	0.267
Previous antibiotic exposure, n (%)	7 (58.3)	13 (65.0)	0.724
Days of treatment, median (range)	12.5 (5–21)	10 (8–13)	0.329
Death, n (%)	3 (25.0)	3 (15.0)	0.647

BTI, biliary tract infection.

**Table 2:** Antimicrobial resistance in *E. coli* isolates causing BTI and non-BTI.

	BTI *E. coli* (n = 15)	Non-BTI *E. coli* (n = 21)	*P*-Value
Ampicillin, n (%)	11 (73.3)	19 (90.4)	0.210
Amoxicillin-clavulanic acid, n (%)	4 (26.6)	6 (28.6)	1.000
Piperacillin-tazobactam, n (%)	2 (13.3)	1 (4.7)	0.559
Cefuroxime, n (%)	1 (6.6)	3 (14.3)	0.626
Cefotaxime, n (%)	0 (0.0)	2 (9.5)	0.500
Ceftazidime, n (%)	0 (0.0)	2 (9.5)	0.500
Cefepime, n (%)	0 (0.0)	2 (9.5)	0.500
Imipenem, n (%)	0 (0.0)	0 (0.0)	1.000
Meropenem, n (%)	0 (0.0)	0 (0.0)	1.000
Ciprofloxacin, n (%)	2 (13.3)	7 (33.3)	0.252
Levofloxacin, n (%)	2 (13.3)	7 (33.3)	0.252
Gentamicin, n (%)	1 (6.6)	2 (9.5)	1.000
Tobramycin, n (%)	1 (6.6)	2 (9.5)	1.000
Amikacin, n (%)	0 (0.0)	0 (0.0)	1.000
Cotrimoxazole, n (%)	4 (26.6)	6 (28.5)	1.000
Tigecycline, n (%)	0 (0.0)	0 (0.0)	1.000
Colistin, n (%)	0 (0.0)	0 (0.0)	1.000
ESBL, n (%)	0 (0.0)	2 (9.5)	0.500
MDR, n (%)	2 (13.3)	5 (23.8)	0.674

BTI, biliary tract infection; ESBL, extended spectrum beta-lactamase; MDR, multidrug-resistant.

**Table 3:** Typing of 15 *E. coli* isolates by multilocus sequence typing.

Strain	Source	*adk*	*fumC*	gyrB	*icd*	*mdh*	*purA*	*recA*	ST	ST Complex
**1-HE**	Blood	6	346	12	1	20	13	7	2230	23
**43-HE ^^^**	Blood	6	4	4	16	24	8	14	58	155
**47-HE**	Blood	6	95	15	18	9	8	6	Unknown	-
**140-HE** *	Blood	53	40	47	13	36	28	29	131	131
**23-AE** *	bile	53	40	47	13	36	28	29	131	131
**3-AE**	Bile	13	6	15	13	16	10	122	Unknown	-
**4-AE ^^^**	Bile	6	4	4	16	24	8	14	58	155
**8-AE**	Bile	6	23	3	16	9	8	6	3640	-
**16-AE**	Bile	10	11	4	8	8	8	2	10	10
**24-AE**	Bile	13	13	9	13	16	10	9	12	12
**60-AE ^~^**	Bile	112	11	5	12	8	8	86	542	-
**61-AE ^~^**	Bile	112	40	4	12	8	8	86	Unknown	-
**66-AE**	Bile	21	35	27	6	5	5	4	69	69
**81-AE**	Bile	13	52	10	14	17	25	17	141	-
**82-AE**	Bile	6	4	14	16	24	8	14	155	155

^^,^* *E. coli* isolated from blood and bile samples of the same patient. *~ E. coli* isolated from bile of the same patient. ST: sequence type.

**Table 4:** Virulence genes from *E. coli* causing biliary tract infection.

Strain	1-HE	43-HE ^^^	47-HE	140-HE *	23-AE *	3-AE	4-AE ^^^	8-AE	16-AE	24-AE	60-AE ^~^	61-AE ^~^	66-AE	81-AE	82-AE
**Source**	Blood	Blood	Blood	Blood	Bile	Bile	Bile	Bile	Bile	Bile	Bile	Bile	Bile	Bile	Bile
***iss***	+	+				+			+	+		+	+		+
***ipfA***	+	+	+			+	+	+				+	+		+
***mchC***						+				+				+	
***mchB***						+								+	
***mchF***	+	+				+				+			+	+	+
***iroN***	+	+				+				+		+	+	+	+
***tsh***	+														
***gad***	+	+	+	+	+		+	+	+	+	+	+		+	+
***astA***			+									+	+		
***senB***									+	+		+			
***iha***										+		+			
***air***													+		
***ailA***													+		
***mcmA***										+				+	
***vat***										+				+	
***ireA***										+					
***papG***	+												+		
***sfa***	+	+	+	+	+	+		+	+	+	+	+	+	+	+
***fimH***	+	+	+	+	+	+	+		+	+	+	+	+	+	+
***iutA***	+	+		+					+	+		+	+		+
***ipaH***													+		
***cnf1***										+					
***hlyA***															
***cdtB***															
***stx-1***															
***stx-2***															
***eaeA***															
***bfp***															
**Total**	10	8	5	4	3	8	3	3	6	14	3	10	12	9	8

*iss:* increased serum survival, *lpfA*: long polar fimbria, *mchC*: microcin H47 maduration system, *mchB*: microcin H47, *mchF*: ABC transporter protein, *iroN:* enterobactin siderophore receptor protein, *tsh*: temperature-sensitive hemagglutinin, *gad:* glutamate decarboxylase, *astA*: heat-stable enterotoxin-1, *senB*: enterotoxin, *iha*: irgA homologue adhesion, *air*: enteroaggregative immunoglobulin repeat protein, *eilA*: Salmonella hilA homologue, *mcmA*: microcin M, *vat*: vacuolating autotransporter toxin, *ireA*: siderophore receptor, *papG*: P fimbrial adhesin, *Sfa*: S fimbrial adhesin, *fimH*: type 1 Fimbriae, *iutA*: ferric aerobactin receptor, *ipaH*: invasion plasmid antigen, *cnf1*: 4cytotoxic necrotizing factor-1, *hlyA*: hemolysin A, *cdtB*: cytolethal distending toxin, *stx-1* and stx-2: Shiga toxins-1 and -2, *eaeA*: intimin, and *bfp*: bundle-forming pilus. *^*^,^* E. coli isolated from blood and bile samples of the same patient. *~ E. coli* isolated from bile of the same patient.

**Table 5:** Resistance genes from *E. coli* causing biliary tract infection.

Strain	Source	*strA*	*bla*_TEM_	*catA*	*sul*	*tet*	*dfrA*	*aph*	*gyrA*	*parC*	*parE*	*aadA*	*mph*
**1-HE**	Blood	+	1a					+					
**43-HE** ^^^	Blood		1b										
**47-HE**	Blood		1a					+				+	
**140-HE** *	Blood		1b		+		+				+(I529L)	+	+
**23-AE** *	Bile		1b		+		+				+(I529L)	+	+
**3-AE**	Bile	+	1b	+	+	+	+						
**4-AE ^^^**	Bile								+(S83L)				
**8-AE**	Bile												
**16-AE**	Bile		1b		+			+					
**24-AE**	Bile												
**60-AE ^~^**	Bile		1b						+(S83L)				
**61-AE ^~^**	Bile				+		+	+	+(S83L)		+(I529L)	+	
**66-AE**	Bile		1b		+	+		+					
**81-AE**	Bile												
**82-AE**	Bile		1b	+	+	+	+	+	+(S83L) (D87N)	+(S80I)		+	

^^,^* E. coli isolated from blood and bile samples of the same patient. *~ E. coli* isolated from bile of the same patient. 1a and 1b: two different *bla*_TEM_ genes.

## Supplementary Materials

The following are available online at https://www.mdpi.com/2077-0383/8/12/2118/s1, Table S1: Typing of the 21 non-biliary *E. coli* isolates by MLST. The results of the typing of the 21 non-biliary *E. coli* isolates by MLST has been included in the supplementary material as Table S1.
