# Supplementary material for: Phylogeny, Resistome, and Virulome of Escherichia coli Causing Biliary Tract Infections
Source: J Clin Med. 2019 Dec 2;8(12):2118. doi: 10.3390/jcm8122118 (PMC6947626; doi:10.3390/jcm8122118)
Supplement: Supplementary file 1 [file jcm-08-02118-s001.pdf]

Supplementary data

Table S1. Typing of the 21 non-biliary *E. coli* isolates by multilocus sequence typing.

Isolate	Source (Focus)	*adk*	*fumC*	gyrB	*icd*	*mdh*	*purA*	*recA*	ST	ST Complex	Phylogroup
**135-HE**	Blood (UTI)	10	11	4	8	8	8	2	10	10	A
**5-HE**	Blood (UTI)	97	40	93	13	23	28	66	429	-	-
**12-HE**	Blood (UTI)	36	24	9	13	17	11	25	73	73	B2
**38-HE**	Blood (UTI)	40	13	9	13	16	10	9	493	12	-
**2-HE**	Blood (UTI)	37	38	19	37	17	11	26	95	95	B2
**272-HE**	Blood (IA)	13	38	84	13	17	64	34	569	-	-
**29-HE**	Blood (IA)	53	40	47	13	36	28	29	131	131	B2
**15-HE**	Blood (Unknown)	37	38	19	37	17	11	26	95	95	B2
**23-HE**	Blood (Unknown)	14	14	10	200	17	7	10	1193	-	-
**31-HE**	Blood (Unknown)	37	38	19	37	17	11	26	95	95	B2
**7-HE**	Blood (CNS)	53	40	47	13	36	28	29	131	131	B2
**118-HE**	Blood (Endocarditis)	53	40	47	13	36	28	29	131	131	B2
**11-HE**	Blood (Endovascular)	10	11	4	8	8	8	2	10	10	A
**21-HE**	Blood (Catheter)	13	43	9	37	17	37	25	567	-	-
**3-HE**	Blood (RTI)	53	40	47	13	36	28	29	131	131	B2
**18-HE**	Blood (endometritis)	92	4	87	96	70	58	2	648	648	-
**33-HE**	Blood (chorioamnionitis)	53	40	254	13	36	28	29	1982	131	-
**74-HE**	Blood (Surgical wound)	43	41	15	18	11	7	6	101	101	B1
**47-AE**	IA abscess	13	38	84	13	17	64	34	569	-	-
**49-AE**	Peritoneal fluid	21	35	27	6	5	5	4	69	69	D
**54-AE**	Peritoneal fluid	21	35	27	6	5	5	4	69	69	D

HE, Hemoculture *E. coli*, ST, sequence type, UTI, urinary tract infection; IA, intra-abdominal; CNS, central nervous system; RTI, respiratory tract infection.
